# Steps towards XAFS beamline automation and remote access

**DOI:** 10.1107/S1600577518007920

**Published:** 2018-06-26

**Authors:** Santiago José Alejandro Figueroa, Douglas Bezerra Beniz, Junior Cintra Mauricio, James Rezende Piton, Stephen A. Parry, Giannantonio Cibin

**Affiliations:** a Brazilian Synchrotron Light Laboratory (LNLS), Brazilian Center for Research in Energy and Materials (CNPEM), Rua Giuseppe Máximo Scolfaro, 10000/PO Box 6192, Campinas, Sao Paulo 13083-970, Brazil; b Diamond Light Source, Harwell Science and Innovation Campus, Diamond House, Didcot, Oxfordshire OX11 0DE, UK

**Keywords:** beamline remote access, beamline automation, XAFS, LabWeb, science studio

## Abstract

Although remote access to beamline synchrotron facilities is now a common operation mode at macromolecular crystallography beamlines thanks to substantial efforts in automated processes for sample preparation and handling, experiment planning and analysis, this is still not the case for XAFS beamlines. Here the experience and developments undertaken at LNLS and Diamond in automation are described, in an attempt to tackle the specific challenges posed by the high variability in experimental conditions and configurations that XAFS measurements require.

## Abstract 2

Remote access to XAFS beamlines is an attractive option to reduce costs and provide an access route for educational and collaborative purposes. Any degree of automation of the operations, even if partial and limited to routine procedures, allows simplification of the remote user interface with significant improvement in the overall beamline efficiency. Here, some useful steps towards achieving this goal are introduced and the experience in this regard at the XAFS beamlines at LNLS and Diamond is described.

## 1. Introduction   

The rapid progress in synchrotron radiation source technology is now providing brighter and smaller beams at new facilities such as Sirius (Liu *et al.*, 2014 ▸) and MAX IV (Eriksson *et al.*, 2013 ▸), while several third-generation sources (ESRF, APS, Diamond, SLAC, *etc*.) are in the process of evaluating, designing or planning upgrades in the same direction. To fully benefit from the improved source characteristics, such enhancements require advances in the development of new detectors and more efficient optical systems. Therefore, it can be expected that, as occurred following the step from second- to third-generation facilities, a significant fraction of the standard experiments that now require long acquisition times and dedicated sessions could be planned in shared beamtimes and will benefit from remote access mechanisms which will maximize efficiency. In turn, this gain in efficiency will require a better organization of research and support teams as well as improved experimental planning and management.

Some research techniques present a natural tendency to be almost entirely automated and then remotely operated. Macromolecular crystallography beamlines are an obvious example (McPhillips *et al.*, 2002 ▸; Leslie *et al.*, 2002 ▸; Smith *et al.*, 2010 ▸; Gabadinho *et al.*, 2010 ▸):

(i) Samples can be well conditioned in standard cryogenic holders.

(ii) Robotic sample changers are available and the technology is now well established.

(iii) The sample alignment procedures work in well defined conditions, and image recognition software has benefitted from substantial development in recent years.

(iv) Thanks to the adoption of large two-dimensional detector systems, the experimental procedure has been greatly simplified: collect one or more images, determine the crystal lattice, measure Bragg peaks and angles, calculate the crystal lifetime, calculate the strategy and data collection time, feed in beam parameters, *etc*.

(v) The data evaluation and analysis chain is reliable and well developed.

This means that, with few exceptions, all MX beamlines now include the possibility of remotely controlled experiments, or even practically dispense with human operation [for example MASSIF-1 at ESRF (Bowler *et al.*, 2013 ▸)] and users just need to send their labelled samples by a courier service.

In the case of other techniques, particularly those based on X-ray absorption and fluorescence, which are the focus of this article, complete automatization is not always straightforward, especially for experiments that involve toxic, flammable or explosive gases, or extreme-condition sample environments (*e.g.* high pressure, high and low temperatures). Further complications arise from the necessity for XAS instruments to cover as wide an energy range as possible, requiring the use of different optical configurations and maintaining flux and beam position stability while scanning energy to collect high-quality data. The variability of the optical configurations needed to optimize the beam for the experiments (*e.g.* beam size, harmonic rejection) can add further complexity to the adjustments required. Therefore, remotely conducting a successful experiment on a XAS beamline requires intervention on a complex set of experimental parameters. The remote access tools should provide clear and immediate feedback on the beamline performance and quality of the experimental data, enabling the operator in charge of the experiment to make decisions based on partial results. The role of automation is therefore to simplify the experimental procedures, minimize the information that needs to be conveyed to the remote operator and guarantee constant instrument quality.

With remote access, users and organizations benefit from a substantial economy in resources (starting from a simple reduction in travel costs to the simplification of logistics, and enabling teams in different locations to participate in the data acquisition and evaluation). In addition to the overall improvement in beamline throughput, the availability of remote access has impacted other important areas, as it allows for better training of new users and opens up opportunities for collaborations between geographically dispersed teams.

To implement a successful remote access mode to XAFS beamlines, several steps towards the automation of optical components, detector optimization and data-acquisition processes are necessary (Oji *et al.*, 2012 ▸; Figueroa *et al.*, 2016 ▸). It is not realistic to envisage a generic solution to this aim, considering the different optical designs, hardware platforms and software solutions adopted at XAFS beamlines. However, numerous steps of the measurement process (including sample preparation, beamline alignment, edge changes, detector optimization, *etc.*) are common to all such beamlines and can be automated. This development has a clear impact not limited to the remote access mode; the algorithms for optimization of the optics and detection systems can be immediately applied to conventional ‘local access’ experiments. Automation can reduce set-up times, minimize the possibility of human error and aid the implementation of a systematic recording of beamline settings and performance.

On the user interface side, developing the software with remote access in mind requires particular attention to the user experience. Graphical interfaces must to be intuitive and easy to operate for different types of users with dissimilar experiences and backgrounds, therefore the software needs to be effective and streamlined. At a lower level, allowing for automatic adjustments means having undertaken a full characterization of the beamline behaviour. In a remote access session, it is not realistic to assume the direct training, assistance and feedback that the on-site beamline scientist can provide to help understand and overcome the interface limitations and ‘quirkiness’ common in codes under continuous development. For these reasons it is extremely important to develop robust codes with no errors or bugs that will hinder the remote user. All the effort devoted to achieving this aim in the codes gives immediate results in terms of flexibility, robustness and substantially improved efficiency necessary for future developments in beamline automation or remote access mode.

In the case of B18 beamline, there are some experimental sessions devoted to groups of users who apply jointly for beamtime spread over a period of two years at a time; this usually consists of a number of researchers from the same research institute or of researchers from smaller institutes in the same region of the UK [Block Allocation Group (BAG)]. These proposals often host a high number of short experiments, each consisting of measurements of a limited number of samples mounted on large capacity sample holders. The automation allows for non-expert users, coming from a large number of institutions participating in the BAG consortia, to access the beamline for a short period and provide a high efficiency for the BAG visits.

Automated procedures help in addition to pursue an intense program of rapid-access experiments, usually run by the beamline staff, which is very effective scientifically and supports a very broad range of science areas. These beamtime access requests are limited to a restricted number of measurements not requiring special sample conditions. The samples delivered to Diamond for the rapid access sessions can be grouped for overnight measurement sessions or scheduled during beamline commissioning days.

The Brazilian synchrotron (LNLS) started providing users remote access to several beamlines some years ago with the LabWeb project (Sherry *et al.*, 2012 ▸; Slepicka *et al.*, 2015 ▸). This project includes beamlines with a high demand such as SAXS1, XAFS1 and XRD1, but this development was limited to delivering access to standard characterization experiments and the user community usually demands more complex experiments. In this sense, this contribution is dedicated to show the steps that were followed to achieve a high automation level on XAFS beamlines that could finally permit beamline remote access to all possible experiments.

## 2. Automation process: instrumentation challenges   

The automation of the beamline setup is the core process allowing remote access. We start from the choice of the edge energy of the element of interest and the experimental conditions. This defines first the beamline optical setup (position of motors, slits, mirrors, energy resolution, harmonics rejection, *etc.*) and then the experimental setup (sample preparation and positioning, ion chamber filling, setting detectors, setting preamplifiers, *etc*.) for the specific measurement.

The implementation of these basic steps depends on the detailed technical configurations adopted on individual beamlines. In the following sections we will describe the different approaches whilst trying to highlight the commonalities found on two beamlines, based on different facilities: XAFS2 at LNLS and B18 at Diamond Light Source.

### 2.1. XAFS2 beamline optical setup automation   

The implementation of the automation process on XAFS2 (Figueroa *et al.*, 2016 ▸) was boosted with the advent of the Sirius project (the new Brazilian synchrotron light source), as some beamlines were deactivated to external users [particularly XAFS1 (Tolentino *et al.*, 2016 ▸)] in order to re-allocate staff to the development of the new beamlines. As a result of the high demand and short time available for external users, it was necessary to automate some beamline processes in order to streamline the experimental setup. We designed a system for automatic filling of gases used in the ionization chambers, an auto-gain adjustment of the ion chamber amplifier electronics, integrating the option to run software macros with several samples and different scan settings (for transmission mode). We are currently developing a procedure for an automatic energy calibration (https://github.com/juniorcmauricio/XAFS2_LNLS) in parallel with other algorithms aimed at reducing the time needed for optimizing the beamline optics: principally mirror adjustment, crystal monochromator parallelism checks and harmonics rejection mechanisms.

#### 2.1.1. Setting crystal monochromator parallelism   

To maximize the photon flux provided over the whole energy range, it is necessary to adjust the double-crystal monochromator (DCM) parallelism. We used the Py4Syn (Slepicka *et al.*, 2015 ▸) library to automate the process. After moving to the selected energy, the script scans the pitch motor of the second crystal, collecting the rocking curve data. The process finds the first maximum of the rocking curve and positions the pitch motor in this position, thus optimizing the parallelism of the crystals, which provides the maximum flux at the selected energy. To maintain the optimal alignment over the whole energy range we are currently developing a feedback system based on a lock-in approach (Proux *et al.*, 2006 ▸; Prestipino *et al.*, 2011 ▸) using a piezoelectric actuator. The main concern here is to be fast enough to detect the tiny changes in *I*_0_.

#### 2.1.2. Setting mirrors   

Depending on the experimental requirements, it can be necessary to move the mirrors to perform a harmonic rejection, to collimate the beam or to achieve a better energy resolution. In our usual configuration, there is no significant harmonic contribution for energies higher than 6 keV because of the cut-off from the Rh mirrors usually positioned at an incidence angle of 3.9 mrad. Movements are then limited to achieve higher scan energies and/or to obtain a certain experimental resolution required for the experiment. These special cases are in the process of being automated by recording the optical-component positions for each configuration and moving the motors to these positions when required.

#### 2.1.3. Harmonic rejection   

For energies lower than 6 keV at XAFS2, we usually detune the second DCM crystal to perform harmonics rejection, maintaining the cut-off angle of the mirrors. This process is usually performed manually by the local contact at the beamline, but, as a straightforward procedure, it is in the process of being automated using a Python script and Py4Syn.

### 2.2. B18: beamline configuration archive and retrieval system   

As a result of the wide energy range requested by the UK XAS community (B18 covers experiments from 2 to 35 keV), the optical design adopted on B18 (Dent *et al.*, 2013 ▸) includes eight (main) different combinations of optical elements. Two mirror branches (with Cr and Pt coatings) allow measurements avoiding absorption edges, and the DCM is equipped with Si(111) and Si(311) crystal pairs, which can be used with both mirror coating positions. To cover the low-energy regimes, harmonic rejection mirrors are inserted to remove the high-energy components. In addition to these combinations, we can tune the focusing-mirror grazing-incidence angle and toroid bend radius to obtain several focus footprints at the sample position. Changing between these configurations requires moving three sets of slits, a translation of the DCM crystal with optimization of its pitch and roll axes, repositioning of the focusing mirror (pitch, yaw alignment and bend radius), insertion or removal of the harmonics rejection mirrors (with optimization in height and pitch) and motion of the experimental table with five degrees of freedom (pitch, roll, height, yaw and horizontal translation).

**Figure 1:**
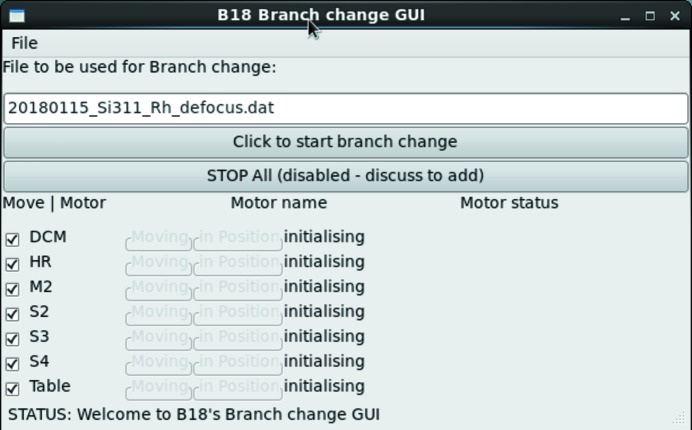
Graphical interface to the optics configuration tool developed on B18.

Each configuration is recorded in a simple database, collecting the position of all the relevant EPICS (Dalesio *et al.*, 1994 ▸) process values (PV) representing motor positions. This set is checked at each beamline start-up, and adjusted after each maintenance intervention on the optics components. Retrieval of these configurations is performed automatically using a code developed in Python that controls the motion of the predefined motor axes in a defined sequence and checks the status of each motion axis. Axes are grouped so the motion of each optical component can follow different strategies adapted to the specific needs of each optical component. For example, to adjust the focusing-mirror axes backlash compensation movements must be applied; whereas for the experimental table, that needs to move approximately 60 mm in the horizontal direction, high tolerance on the roll axis must be maintained to prevent damage to the beamline vacuum. Therefore, we use an incremental step motion-and-check strategy. The check associated with each small movement minimizes risks associated with possible clashes of the table with nearby devices installed in the experimental hutch, as discrepancies between requested and achieved positions will highlight the presence of potential interference with the table motion. The whole configuration retrieval sequence is programmed to occur in parallel on all the optical systems, so a full-beamline-configuration change can take less than a minute. The simple graphical user interface (GUI) (Fig. 1 ▸) layer of the retrieval code reports on the overall status of the motion adjustment steps and allows an immediate identification of issues in the sequence for each optical element.

### 2.3. Experimental-setup automation   

#### 2.3.1. Automatic filling of the ion chambers   

**Figure 2:**
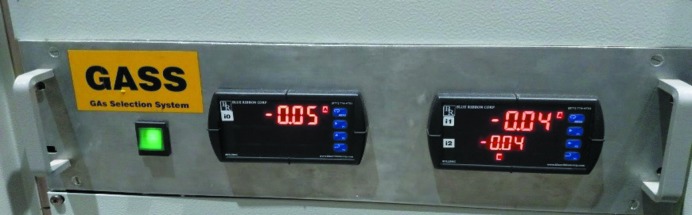
Gas selection system module developed at LNLS.

At XAFS2, for each energy range measured, it is necessary to adjust the gas composition used for the ionization chambers so that the first chamber absorbs 10% intensity of the monochromatic beam and the second chamber absorbs 70%. We use three gases (helium, nitro­gen and argon) at a pressure of 0.5 bar. Historically, the gas exchange was performed manually and the composition was calculated using the *Hephaestus* software (Ravel *et al.*, 2005 ▸). This recurring and time-consuming process was automated (Mangold *et al.*, 2013 ▸). We designed a system based on solenoid valves, pressure-limiting valves, a vacuum system for purging ionization chambers and pressure gauges. A graphical interface performs IOC communications with the equipment [gas selection system (GASS), shown in Figs. 2 ▸ and 3 ▸], which in turn automatically adjusts the gas mixture inside the ionization chambers.

**Figure 4:**
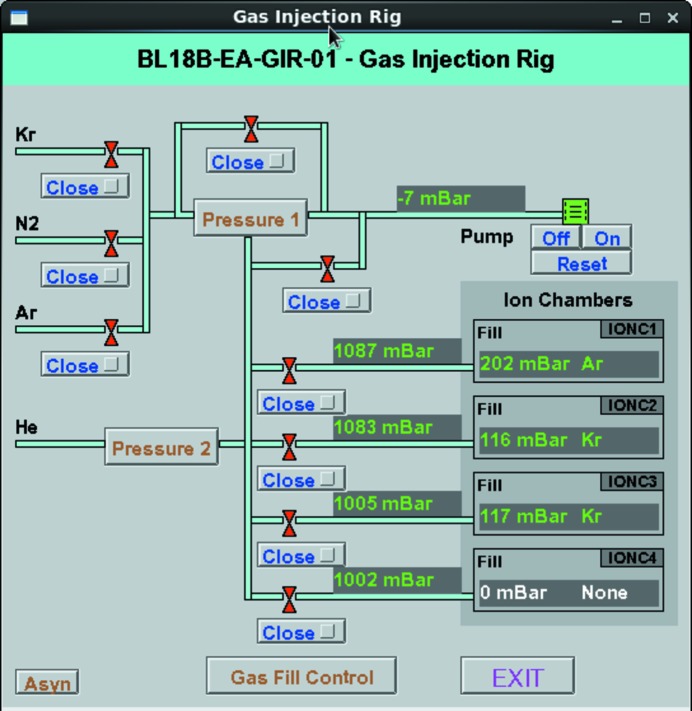
B18 EPICS EDM panel for controlling the gas filling in the ionization chambers.

On B18, the ion-chamber gas-fill system feeds the three ionization chambers and an additional feedline is available both as a spare in case of failures and if experimental devices need inert gas pressure control. Low-level control and serial communication is implemented with a PLC unit, and the EPICS layer (Fig. 4 ▸) is integrated with the general-beamline vacuum IOC. In addition to giving manual access to controlling the vacuum pump, valves, pressure gauges and pressure controllers (pressure control units from ALICAT), the whole fill sequence (including control of the high-voltage power supplies) is programmed in the EPICS layer. Purging is obtained by pumping until pre-defined low-pressure thresholds are reached, or timeouts occur. An optional flush with He can be performed by the system to remove traces of highly absorbing gases when moving the configuration to lower energies.

**Figure 5:**
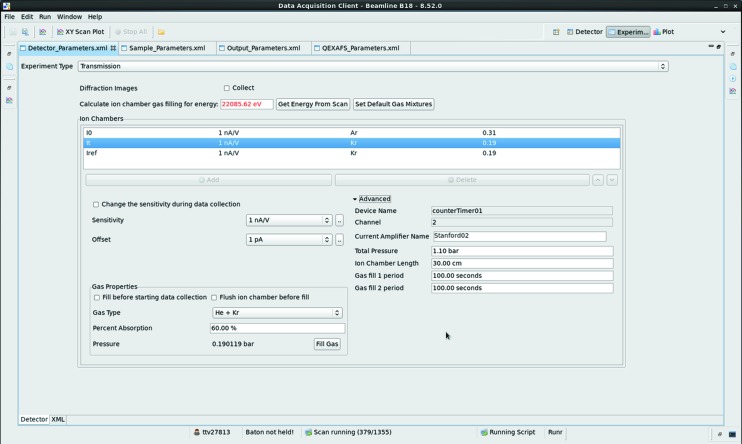
The detector configuration panel in the *GDA* experimental setup for XAS experiments.

The gas fill can be programmed using *Generic Data Acquisition* (*GDA*), the open-source framework data-acquisition software developed at Diamond. For the spectroscopy beamlines, each XAS measurement is associated with a set of data-acquisition-configuration files controlling the experimental parameters (filename, detector, scan speed, data resolution, *etc.*). The detector configuration file (an xml file generated by the experiment GUI and interpreted at runtime by the data-acquisition server, see Fig. 5 ▸) contains appropriate fields specifying the desired gas partial-fill pressures and the filling-sequence parameters. Each configuration file is associated with each sample, allowing the ion chamber filling to be automatically changed while running a sequenced queue of samples. For initial setup of this file, the user is supported in the GUI environment with a suggested default choice of gas and partial pressures for gas filling, calculated from the energy range selected for the XAS measurement (stored in a scan parameter xml file), and reports the estimated absorption corresponding to the chosen mixture.

#### 2.3.2. Auto tuning the ion chambers   

Users at XAFS2 now have access to a new software tuning tool in order to automatically select the appropriate gain in the current pre­amplifiers (Stanford SR570 units). The tuning tool performs a scan on all available gains in the preamplifier, saving the results in a vector and setting the gain corresponding to the result closest to the ideal output (around 3 V for each ion chamber).

On B18, each amplifier setting can be programmed in the same *GDA* detector setup configuration file used for programming the gas filling. Currently, automatic amplification adjustment is run by calling a separate script (integration within the overall GUI is foreseen for the next *GDA* release), which adjusts the output voltage so maximum amplification is obtained with the sample and a reference foil (placed between the second and third ion chamber) in the beam. Amplification levels are checked at two energies, a few eV before the absorption edge and at the end of the scan to avoid saturation of the amplifiers across the whole XAS scan energy range.

#### 2.3.3. Calibration issues   

For transmission experiments at XAFS2, we have developed an edge-energy calibration automation procedure that performs the scanning of a metallic foil of the corresponding element. The procedure identifies the first derivative of the acquired spectrum, finds the first maximum (first inflection point), positions the theta motor in this position and performs, if necessary, the calibration energy, assigning an offset value to the theta-motor position. On B18 this procedure is not yet automated, as, once a full beamline configuration change is completed, the calibration remains sufficiently accurate over a large angular range for the DCM. However, a large wheel array of metal foils and reference compounds is permanently installed in a He-filled vessel placed between the second and third ionization chamber, and is used for a simultaneous acquisition of an energy-calibration scan for each transmission experiment. For experiments in fluorescence or total electron yield, we can obtain a reference sample measurement using part of the low-angle diffuse scattering from the last window of the first ionization chamber, and reading the fluorescence (or transmitted beam) from the foil placed close to main beam path.

## 3. Remote access experiments: a remote controlling approach of XAFS2 using LabWeb at LNLS   

**Figure 6:**
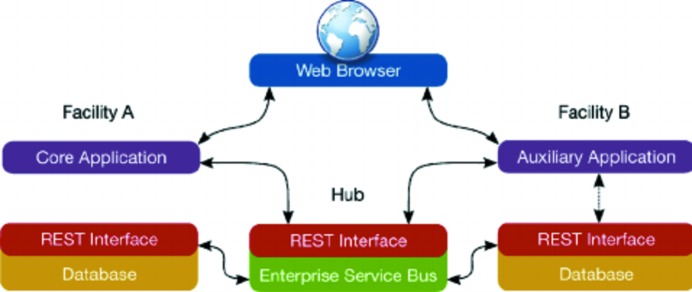
Schematic of the services used by LabWeb for remote access and control of parts of the XAFS2 beamline at LNLS, based on Science Studio (Sherry *et al.*, 2012 ▸).

Some years ago, the Brazilian and Canadian light source laboratories, in a joint effort, implemented a solution to remote operation of a beamline, which is known as LabWeb (Fig. 6 ▸) (Slepicka *et al.*, 2013 ▸).

**Figure 7:**
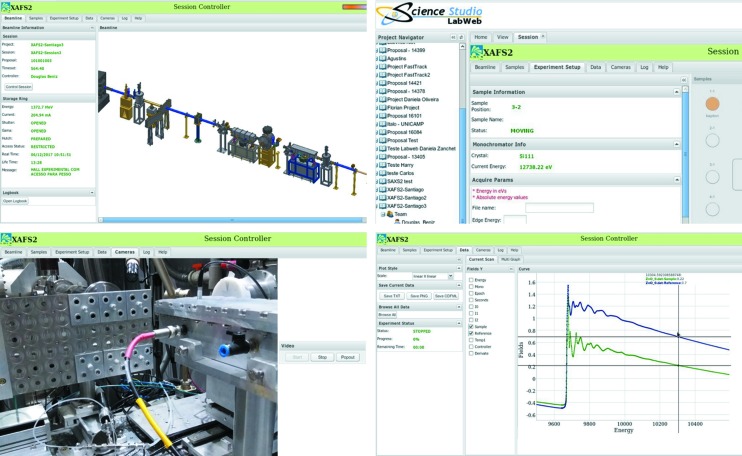
Four selected screenshots of XAFS2 implementation of LabWeb: beamline status, experiment setup, monitoring cameras and experiment results.

Initially, this project aimed to cover only the most straightforward experiments. An important limitation to the experimental flexibility was the result of safety considerations. In particular, remote access was considered not feasible for *in situ* or *operando* experiments, which often require controlling high-temperature furnaces and mass-flow controllers of dangerous atmospheres. An important aspect to consider is the wide area network reliability. At the moment, a limited number of users are expected to participate in remote access experiments on a small number of beamlines, so the network infrastructure is robust enough to guarantee that full functionality is available to the operators, and beamtime is used efficiently. However, the consequences of unstable or slow network conditions were considered, so the acquisition system is programmed to take predefined actions in the case of an extended period without contact with the master operator of the experiment (Fig. 7 ▸).

To fully support XAFS2 users, a project was started to improve the capabilities of LabWeb. As described in the LabWeb project paper, server modules are now implemented using Java technology. For device control, we adopted the model of the EPICS control system and its concept of a distribution system with a server engine interacting with the control system platform of the experimental hutch (Mangold *et al.*, 2013 ▸). At the end of the first development phase, the implementation of LabWeb allows authorized users, within a predefined scheduled time, to operate the XAFS2 beamline remotely with control of the standard devices which are considered safe to start remotely. Such operation includes the ability to: control network cameras to visualize the hutch internally and externally; inspect the beam shape and size; control the sample-manipulation area; configure an acquisition sequence and corresponding sample specific identification; start and monitor the acquisition progress; visualize the experimental results in the form of raw and processed data; transfer the data to the home institution at the end of a set of experiments.

In the second phase of this project, we intend to extend the automation to give to remote users the possibility of controlling furnaces, cryojets and mass flow controllers. This is expected as an advancement in Sirius operation with improvements to safety that will come in the form of a closed experimental hutch with a controllable atmosphere that can minimize the risk of fire and extract toxic gases. Following this aim, we are revising all technologies used in LabWeb which is based on Java, and considering the opportunities offered by more flexible programming frameworks like Python. An option under evaluation is moving to *MXCuBE*3 (Mueller *et al.*, 2017 ▸), a platform designed for macromolecular crystallography beamlines. This will offer a more modern approach for using web interfaces in experimental control systems as it natively supports use from personal devices (*e.g.* tablets and smartphones).

## 4. Perspectives and concluding remarks   

Our main objective in this contribution is to highlight, based on the experience gained at LNLS and Diamond, both the challenges posed and the immediate benefits offered by the development of automated methods and remote access systems. Beyond the differences in hardware and software solutions implemented, different levels of automation and user community requirements, we find that common strategies, aims and areas for development have emerged and are driven by the continuous interaction with our evolving user communities. Development of consistent and self-explanatory software interfaces, associated with a robust level of automation, is essential to improve the efficiency of beamtime sessions open to new and inexperienced users, and to allow successful implementation of web-based remote control systems.

## Figures and Tables

**Figure 3:**
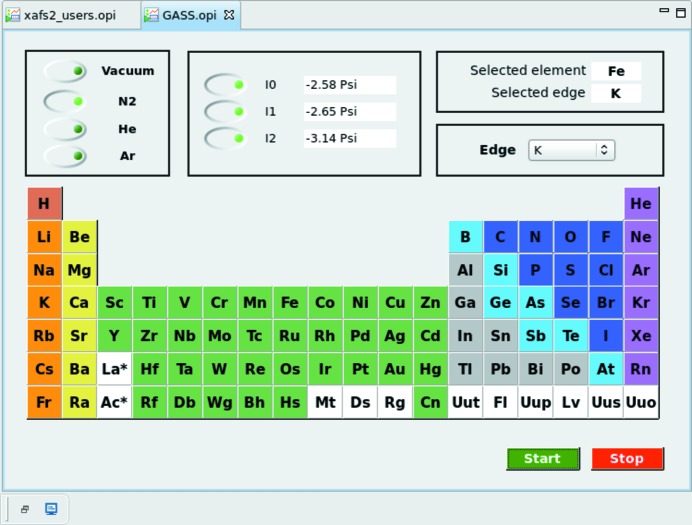
XAFS2 panel for controlling the gas filling in the ionization chambers.
